# Synthesis, spectroscopic study, antioxidant, DNA-cleavage and enzyme-mimicking activities for metal^(II/III)^ complexes with azo ligand–eugenol scaffold

**DOI:** 10.1098/rsos.252298

**Published:** 2026-10-21

**Authors:** Huda Muayad Nafea, Abbas Ali Salih Al-Hamdani

**Affiliations:** ^1^ Department of Chemistry, College of Science, University of Baghdad, Baghdad, Iraq; ^2^ Department of Chemistry, College of Science for Women, University of Baghdad, Baghdad, Iraq

**Keywords:** anthraquinone, antioxidant, DNA cleavage, eugenol, phenoxazinone synthase activity

## Abstract

A newly synthesized azo ligand, 1-((5-allyl-2-hydroxy-3-methoxyphenyl)diazenyl)anthracene-9,10-dione (H-MND), derived from an anthraquinone nucleus coupled with the natural phenol (eugenol), was employed to prepare Co(II), Ni(II), Cu(II), Rh(III) and Pd(II) complexes, with the ligand proposed to adopt a tridentate coordination mode. Anthraquinone derivatives and natural phenols are recognized for diverse pharmacological activities, making them valuable scaffolds for the design of bioactive ligands. The synthesized compounds were characterized by FT-IR, UV–vis, NMR spectroscopy, mass spectrometry, elemental analysis, thermal analysis thermogravimetric-differential scanning calorimetry (TGA-DSC), assessment of chlorine content, magnetic susceptibility and molar conductance measurements, which collectively suggested predominantly non-electrolytic behaviour. In the 1,1-diphenyl-2-picrylhydrazyl (DPPH) assay, the Ni(II) complex exhibited the highest antioxidant activity among the synthesized compounds, with an IC_50_ value significantly different from that of gallic acid (*t* = 4.31, *p* ˂ 0.01). Only the Pd(II) complex displayed activity towards plasmid DNA consistent with partial cleavage in the absence of externally added redox agents. Furthermore, activity mimicking phenoxazinone synthase was evaluated for the Cu(II) and Co(II) complexes, revealing system-specific catalytic behaviour. These results support further investigation into the biomedical and catalytic potential of these metal complexes.

## 1. Introduction

Azo compounds are characterized by two phenyl rings linked by an N═N group. Azo dyes, a specific category, contain one or more aromatic or heterocyclic units that involve at least one conjugated azo chromophore [1–3]. The existence of conjugated and chromophoric systems enables them to display a wide range of colours, making them essentially colourants. As a result, these chemicals have attracted considerable interest from researchers across a wide range of applied and fundamental research fields [4,5]. In medicinal chemistry, a variety of metal chelates are recognized as necessary; significant progress in metal chelate research has drawn interest from the bioinorganic community [6,7]. Transition-metal chelates of azo dyes are essential in coordination chemistry because the increased number of donor atoms in the ligands enhances complexation potential [8–10]. Additionally, crucial natural product derivatives of anthraquinones possess various pharmacological properties, including anti-tubercular, antibacterial and anti-HIV activities, and have beneficial applications in the textile industry [11]. In particular, metal complexes derived from anthraquinones are of vital interest in bioinorganic chemistry for therapeutic development [12–14]. Similarly, eugenol (4-allyl-1-hydroxy-2-methoxybenzene) is an essential phenylpropene and a principal constituent of clove oil. It has a wide range of pharmaceutical benefits, including cardiovascular benefits, antidepressant effects, anti-carcinogenic effects and antioxidant activity, and is used extensively in medicine, flavouring and perfumery [15,16]. Furthermore, eugenol-derived azo dyes synthesized via amine coupling have been shown to exhibit variable antimicrobial efficacy and anti-cancer potential [17,18].

Metalloenzymes are a distinctive class of catalysts that can mediate a wide range of transformations in both biological and chemical systems. In addition to their physiological roles, interactions between metal ions and cellular systems are fundamental to understanding immune responses and toxicological pathways [19]. Their catalytic efficiency has inspired the development of model metal complexes that mimic enzymatic processes in laboratory environments. Among these, transition-metal complexes have garnered considerable attention for their role in catalysing the oxidation of organic substrates, thereby providing valuable insights into biomimetic chemistry [20]. A multicopper oxidase known as phenoxazinone synthase (PHS) catalyses the oxidation coupling of two substituted o-aminophenol (OAP) to generate 2-aminophenoxazin-3-one (APX). This process culminates in the production of actinomycin D [21,22], a therapeutically significant antibiotic used to treat Wilms' tumour and several other malignancies, by *Streptomyces antibioticus* [23]. Recent efforts have focused on replicating PHS action using synthetic complexes derived from first-row transition metals, such as cobalt, manganese and copper [22,24,25]. In addition, the effectiveness of many metallodrugs in biological applications is linked to their ability to cleave DNA through either hydrolytic or oxidative pathways [26,27]. Drawing inspiration from the metal-ion active sites found in natural nucleases, researchers have developed synthetic metal complexes as simpler systems to facilitate metal-dependent DNA cleavage, highlighting their potential for biomedical and anti-cancer applications [28,29]. At the same time, antioxidants are necessary for neutralizing free radicals. When present in excess, free radicals can cause oxidative stress and contribute to diseases such as cancer, diabetes and inflammation [30]. Metal complexes exhibiting antioxidant properties rely on their metal centres and ligands, and several research groups have reported new metal-based antioxidant agents with therapeutic potential [31–33].

Although numerous studies have reported azo metal complexes and their biological applications, research on metal complexes derived from azo-anthraquinone ligands remains relatively limited. Specifically, studies examining the influence of different coordinated metal ions on the biological and catalytic properties of complexes within a common azo-anthraquinone ligand framework are scarce. The present study reports the synthesis and characterization of a new azo-anthraquinone-based ligand, 1-((5-allyl-2-hydroxy-3-methoxyphenyl)diazenyl)anthracene-9,10-dione (H-MND), derived from an anthraquinone-eugenol scaffold, as well as its Co(II), Cu(II), Ni(II), Rh(III) and Pd(II) complexes, with the ligand suggested to adopt a tridentate coordination mode. Both the ligand and its 1 : 1 (metal : ligand) stoichiometric complexes were characterized using a combination of analytical, physicochemical and spectroscopic techniques. Moreover, the antioxidant and DNA cleavage activities were evaluated. The divalent copper and cobalt complexes were also investigated for PHS-like catalytic activity using kinetic studies. The azo-anthraquinone framework was selected to study the effects of various transition-metal ions on the resulting complexes. This research provides insights into how the identity of the metal ion affects the catalytic and biological properties of an azo-anthraquinone ligand platform.

## 2. Experimental section

All chemicals used in this study were sourced from British Drug Houses (BDH), Merck and Fluka products. Elemental microanalyses (C, H, N) were conducted using a Eurovector model EA-3000, single/V-3.O. The NMR spectra were recorded on a Bruker 400 MHz spectrometer in DMSO─d_6_, using tetramethylsilane as the internal standard. An electrothermal apparatus was employed to assign the melting points of the new ligand and its chelates. The metal content was determined using a Shimadzu AA680 flame atomic absorption spectrophotometer. The molar conductance of the prepared complexes at a concentration of 10^−3^m in dimethyl sulfoxide (DMSO) was measured at 25°C using a W-T-W conductivity meter. Magnetic susceptibility measurements were obtained with the MSB-MK-1 model. Mass spectra for all compounds were acquired using a Shimadzu QP50A-DI Analysis QP-2010. Thermogravimetric (TGA) and differential scanning calorimetry (DSC) analyses of the solid compounds were performed in air at a heating rate of 10°C min^−1^ over the temperature range of 25–600°C using a Shimadzu TGA analyser. UV–vis spectra were obtained using a Shimadzu-1800 spectrophotometer with a quartz cell (200–1100 nm). Infrared (IR) spectra were measured using a Shimadzu FTIR-8400S spectrophotometer, utilizing KBr and CsI discs.

### 2.1. Synthesis of 1-((5-allyl-2-hydroxy-3-methoxyphenyl)diazenyl)anthracene-9,10-dione ligand and metal complexes

The method described in [14] was slightly modified to prepare a new ligand. The azo ligand was synthesized by performing a diazotization reaction with 1-aminoanthraquinone and sodium nitrite in sulfuric acid at 0–5°C, followed by coupling the resulting diazonium salt with eugenol in an aqueous sodium hydroxide solution while stirring continuously. The final product was isolated via filtration, washed with distilled water and diethyl ether, and subsequently dried in a desiccator using anhydrous CaCl_2_. ^1^H-NMR (supplementary material, figure S1) (400 MHz, DMSO─d_6_), (*δ*, ppm): 2.69 (s, 3H, Ar─OCH_3_), 6.33–6.38 (m, 1H, CH═CH_2_), 6.49–6.56 (m, 3H, Ar─H), 6.61 (d, 1H, Ar─H), 6.82 (s, 1H, Ar─H), 7.57 (d, 2H, Ar─H), 8.57 (d, 2H, Ar─H), 9.16 (s, 1H, labile H); ^13^C-NMR (supplementary material, figure S2) (100 MHz, DMSO─d_6_), (*δ*, ppm): 22.36, 44,78, 75.78, 78.37, 98.54, 98.67, 98.89, 108.34, 108.54, 108.85, 118.11, 118.26, 119.06, 119.16, 125.31, 125.69, 134.86, 142.26, 160.04, 160.15, 180.30, 186.85, 195.31.

Metal complexes were prepared by reacting equimolar amounts of the ligand and corresponding metal salts (CoCl_2_·6H_2_O, NiCl_2_·6H_2_O, CuCl_2_·2H_2_O, RhCl_3_·3H_2_O and PdCl_2_) in hot absolute ethanol under reflux for the appropriate reaction times. The reaction progress was monitored using thin-layer chromatography. Once the reaction was complete, the precipitated complexes were filtered, washed with hot ethanol and diethyl ether, and dried with anhydrous CaCl_2_ in a desiccator. For the Pd(II) complex: ^1^H-NMR (supplementary material, figure S3) (400 MHz, DMSO─d_6_), (*δ*, ppm): 3.05 (s, 3H, Ar─OCH_3_), 7.58–7.60 (m, 1H, CH═CH_2_), 7.85–7.87 (m, 5H, Ar─H), 8.12–8.15 (m, 4H, Ar─H); ^13^C-NMR (supplementary material, figure S4) (100 MHz, DMSO─d_6_), (*δ*, ppm): 25.97, 45.09, 63.66, 68.72, 75.28, 81.46, 86.87, 88.04, 91.15, 96.28, 105.67, 113.64, 125.93, 127.24, 130.55, 133.51, 135.05, 146.64, 148.28, 151.22, 158.24, 182.99, 189.66.

The preparation methods for the ligand and its metal chelates are detailed in the experimental section of the supplementary material, while table 1 summarizes the isolated yields and physical properties of these compounds.

**Table 1.** Physical characteristics and analytical identification of the azo ligand (H-MND) and its metal complexes.

compound molecular formula (M.Wt)	m.p. (°C) colour	elemental analysis found (calc.)						conductivity Ω^−^^1^ cm^2^ mol^−^^1^
		yield %	C %	H %	N %	M %	Cl %	
H-MND C_24_H_18_N_2_O_4_ (398.42)	185–187 dark plum	74	71.88 (72.28)	4.45 (4.51)	8.17 (7.02)	–	–	–
[Co(MND)Cl] C_24_H_17_ClCoN_2_O_4_ (491.79)	264–267 dark brown	76	58.27 (58.61)	3.21 (3.48)	5.34 (5.70)	12.09 (11.95)	7.69 (7.20)	9
[Ni(MND)(H_2_O)_2_Cl] C_24_H_21_ClNiN_2_O_6_ (527.58)	>300 black	82	55.02 (54.64)	3.86 (4.01)	5.67 (5.31)	12.01 (11.12)	6.15 (6.72)	17
[Cu(MND)Cl] C_24_H_17_ClCuN_2_O_4_ (496.41)	>300 (decompose) dark brown	72	57.78 (58.07)	3.28 (3.45)	5.79 (5.64)	11.98 (12.79)	8.03 (7.14)	13
[Rh(MND)(H_2_O)Cl_2_] C_24_H_19_Cl_2_N_2_O_5_Rh (589.23)	>300 reddish brown	74	49.69 (48.92)	3.99 (3.25)	5.66 (4.75)	18.18 (17.46)	12.69 (12.03)	11
[Pd(MND)Cl] C_24_H_17_ClN_2_O_4_Pd (539.28)	278–281 reddish black	69	53.92 (53.45)	2.98 (3.18)	5.89 (5.19)	19.03 (19.73)	5.87 (6.57)	7

### 2.2. Biological methods (*in vitro*)

All biological experiments were carried out in compliance with standard laboratory biosafety and ethical guidelines.

#### 2.2.1. Antioxidant studies (DPPH method)

The antioxidant activity of both the free ligand and its metal chelates was assessed *in vitro* using the 1,1-diphenyl-2-picrylhydrazyl (DPPH) assay [34] (refer to the supplementary material for the detailed experimental method).

#### 2.2.2. Plasmid DNA-cleavage experiment

The DNA cleavage experiments were conducted using the pTXB1 plasmid (New England Biolabs, Ipswich, MA, USA). For detailed experimental methods, see the supplementary material.

#### 2.2.3. Oxidation of o-aminophenol via catalytic complexes

The phenoxazinone synthase-like activity was evaluated spectrophotometrically under aerobic conditions using OAP as the substrate in DMSO at 27°C. Time-dependent spectral changes were recorded at 436 nm, the characteristic wavelength of the generated phenoxazinone chromophore (APX). Control experiments performed under the same conditions in the absence of a metal-complex catalyst showed no significant increase in absorbance at 436 nm. The initial catalyst concentrations were kept constant at 3 × 10^−5^m for the Cu(II) complex and at 6 × 10^−5^m for the Co(II) complex. Reaction rates were assessed from the initial rates method using linear regression of the initial linear region of the absorbance time plots [35]. For rate calculations, the molar absorptivity of 18 300 m^−1^ cm^−1^ for 2-aminophenoxazine-3-one (APX) formation was utilized [36]. Steady-state kinetic parameters were determined at pseudo-first-order conditions (substrate concentration was maintained at least 10 times the catalyst concentration). The substrate concentration ranges used in the saturation kinetics were 1–12 × 10^−3^m and 0.2–2 × 10^−3^m for Cu(II) and Co(II) systems, respectively. All kinetic measurements were performed in four independent replicates (*n* = 4), and kinetic parameters (*V*_max_, *K*_M_ and *k*_cat_) were obtained by nonlinear regression using the Michaelis–Menten model in GraphPad Prism. Results are reported as mean ± s.d.

## 3. Results and discussion

Azo ligand 1-((5-allyl-2-hydroxy-3-methoxyphenyl)diazenyl) anthracene-9,10-dione (H-MND), and its metal complexes, including Co(II), Cu(II), Ni(II), Rh(III) and Pd(II), have been synthesized, and their synthetic pathways are illustrated in scheme 1. Structural investigations were conducted using a range of physicochemical techniques. All metal chelates are coloured, stable in air, non-hygroscopic, and soluble in common organic solvents. The elemental analysis of the experimental and theoretical findings is in satisfactory agreement with the proposed molecular formulae and is consistent with a 1 : 1 (M : L) ratio in the complexes. The molar conductance (*Λ*_m_) values of the complexes were measured in DMSO (10^−3^m) and are reported in table 1. The conductance values for the non-electrolytes (less than 50 Ω^−1^ cm^2^ mol^−1^) and 1 : 1 electrolytes (50–85 Ω^−1^ cm^2^ mol^−1^) are in accordance with the criteria of Geary for DMSO solutions [37]. The experimental values for these complexes range from 7 to 17 Ω^−1^ cm^2^ mol^−1^. These results support predominantly non-electrolytic behaviour and suggest inner-sphere coordination of the anionic ligands under the given experimental conditions. Furthermore, the metal and chloride contents in the complexes were analysed by atomic absorption and argentometry, respectively. The significant analytical and physical data that were obtained are presented in table 1.

**Scheme 1.**
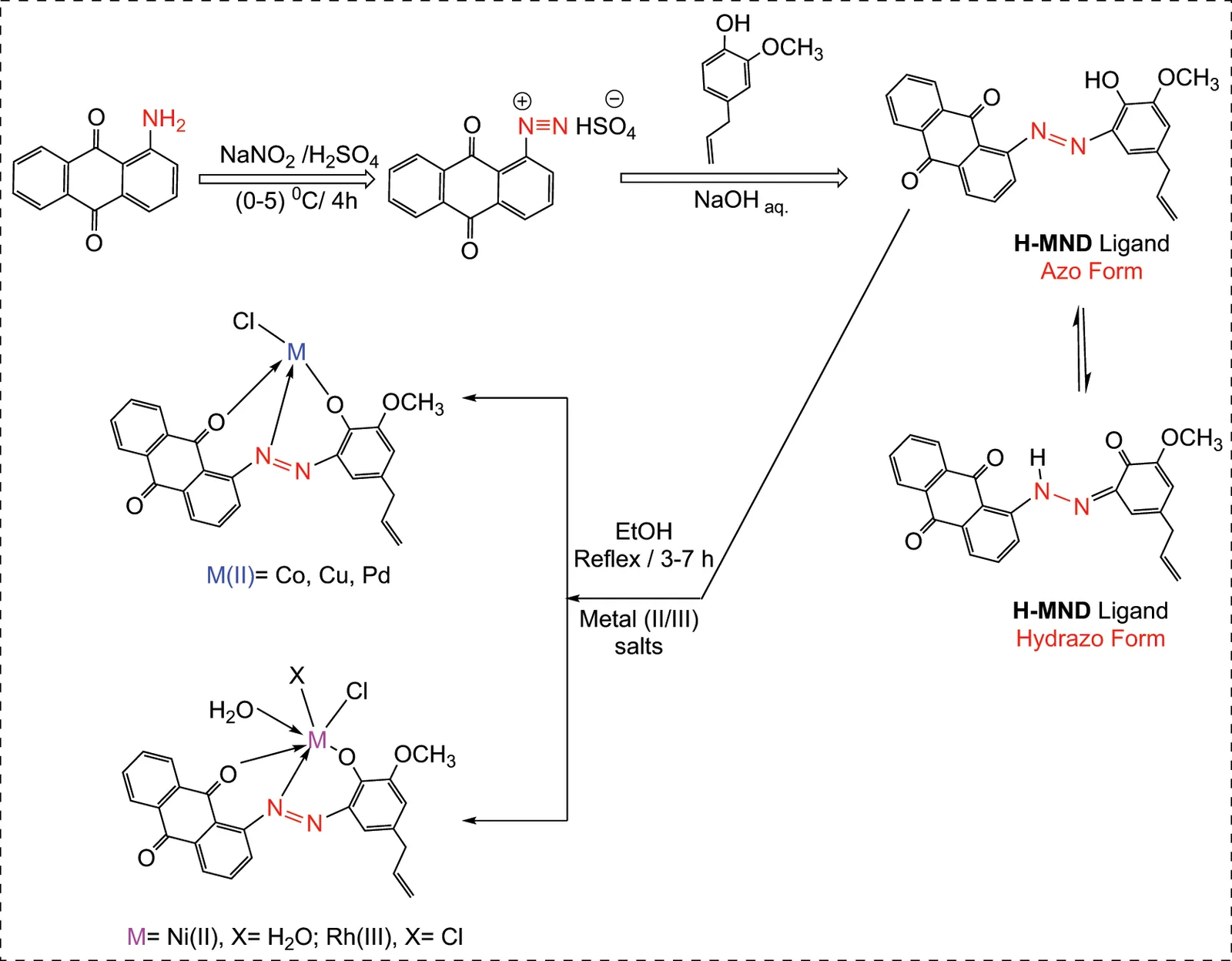
Synthesis diagram of the ligand, showing possible tautomeric forms and proposed structures for its metal chelates.

### 3.1. NMR analysis

In the ^1^H-NMR spectrum of ligand (H-MND), a deshielded labile proton appears as a singlet at *δ* 9.16 ppm (supplementary material, figure S1). By comparison, the hydroxyl (OH) resonance in eugenol is observed at *δ* 5.0–6.0 ppm [38]. The downfield position of this resonance reflects the highly conjugated electronic environment of the azo ligand and suggests the presence of hydroxyazo–ketohydrazone tautomerism in solution. In addition, in the range of *δ* 6.49–8.58 ppm, the aromatic protons of the ligand appear as a collection of multiplets, doublets and a singlet. The aromatic ring of eugenol demonstrates a singlet at *δ* 2.69 ppm for the three (Ar─OCH_3_) protons, and vinyl protons are observed at *δ* 6.33–6.38 ppm [39]. The ^13^C-NMR spectrum of the free ligand (supplementary material, figure S2) displays signals at 195.31 and 186.85 ppm assigned to the carbonyl carbon adjacent to the azo group and the second carbonyl carbon of the anthraquinone moiety, respectively. Another resonance at 180.30 ppm is consistent with the ketohydrazone tautomeric form in solution, in agreement with the labile OH proton detected in the ^1^H-NMR spectrum [40,41]. Upon coordination with Pd(II), the absence of the labile proton signal at *δ* 9.16 ppm in the ^1^H-NMR spectrum of Pd(II) chelate (supplementary material, figure S3) supports the findings of the ^13^C-NMR spectrum of this complex (supplementary material, figure S4), which offers a signal at *δ* 180.30 ppm that shifts to *δ* 151.22 ppm, consistent with deprotonation and formation of a coordinated enolate/phenolate form. The carbonyl signal adjacent to the azo group shifts from *δ* 195.31 to *δ* 182.99 ppm, suggesting involvement in coordination. In comparison, the second carbonyl signal shows only a minor shift from *δ* 186.85 to *δ* 189.66 ppm, implying it remains uncoordinated. The aromatic carbon that is methoxy substituted resonates at *δ* 160.04 ppm in the free ligand and at *δ* 158.24 ppm in the Pd(II) complex. The combined ^1^H- and ^13^C-NMR data support the coordination of the ligand to the Pd(II) centre via the proposed ONO tridentate donor set. For the other complexes, the proposed coordination modes are based on combined IR, UV–vis, magnetic, conductivity and mass spectrometric data, which are consistent with analogous coordination behaviour across the series and are further supported by spectroscopic features observed for the Pd(II) complex.

### 3.2. IR spectral studies

Infrared spectra provide vibrational data that, when compared between the free ligand (H-MND) and its chelates, reveal the coordination mode, as illustrated in table S1 of the supplementary material. Azo ligands commonly feature an ortho-hydroxyl group that promotes hydroxyazo–ketohydrazone tautomerism [42]. In the IR spectrum of the free ligand (supplementary material, figure S5), a broad absorption band at 3421 cm^−1^ is assigned to ν(OH), which is consistent with the proposed tautomeric equilibrium (scheme 1) [43,44]. Chelation through the phenolic oxygen is supported by the disappearance of the phenolic OH (labile proton) signal in the ^1^H NMR spectrum upon complexation, along with the shift of the ν(C─O) band from 1114 to approximately 1170 cm^−1^ in the FT-IR spectra of all complexes. This suggests deprotonation of the phenolic group and possibly reflects the formation of a metal–oxygen coordination bond [45,46]. Relative to the free ligand (1452 and 1469 cm^−1^), the ν(N═N) stretching band in the complexes shifts to a higher frequency, suggesting that the azo group coordinates across the nitrogen atom [47]. By contrast, the bands near 1589 cm^−1^ remain unchanged and are assigned to aromatic ν(C═C) vibrations [48]. The IR spectrum of the ligand exhibits absorption bands at 1676 and 1635 cm^−1^, attributed to the carbonyl groups of the anthraquinone moiety [49]. The higher frequency band is assigned to the carbonyl group that is not directly conjugated to the azo/hydrazone system. The lower frequency band is associated with the carbonyl conjugated to this system. This band may also overlap with the terminal alkene stretching vibration of the allyl substituent (eugenol), typically observed near 1637 cm^−1^ [50]. This overlap makes it challenging to assign the bands in this region unambiguously. Upon complexation, the ν(C═O) band at 1676 cm^−1^ remains largely unchanged, suggesting that this carbonyl group is not directly involved in coordinating with the metal. A new band appears at around 1541–1544 cm^−1^ in all the metal complexes, probably arising from coupled vibrational modes within the delocalized chelate framework, including contributions from C─O, N═N and skeletal ring vibrations. Although these spectral changes align with coordination involving the enolate oxygen and the carbonyl group adjacent to the azo functionality, the IR data alone do not allow for a definitive determination of the coordination mode. However, these observations are in agreement with the coordination mode proposed by the ^1^H and ^13^C NMR data for the Pd(II) complex. Furthermore, new medium-intensity bands were observed in all complexes within the ranges of 580–594 and 437–522 cm^−1^, which are assigned to the ν(M─O) and ν(M─N) stretching vibrations, respectively, based on comparison with reported values in the literature [51]. Additionally, low-intensity bands in the 270–335 cm^−1^ region are attributed to the stretching vibrations ν(M─Cl) in agreement with previous far-IR results for transition metal chloro complexes [43]. Since metal–ligand vibrations may appear as coupled or partially overlapping bands in the low-frequency region, these assignments were made by comparing them with existing literature and general spectroscopic evidence. In the Ni(II) and Rh(III) complexes, bands at 860–889 and 624–655 cm^−1^ correspond to ρ_r_(H_2_O) and ρ_w_(H_2_O), respectively [9,52]. Representative FT-IR spectra of the Co(II) and Pd(II) complexes are supplied in the supplementary material, figures S6 and S7, respectively.

### 3.3. Mass spectrometric studies

Analysing the mass spectra of the uncoordinated ligand and its chelated compounds provides essential insights into their structures. The mass spectrum of the ligand (H-MND) offers a molecular ion peak at *m/z* = 396.24, corresponding to its molecular weight of 398.42 g mol^−1^, with the formula C_24_H_18_N_2_O_4_. The ligand's fragmentation pattern reveals the expected fragmentation pathways and the assigned positively charged fragments, further supporting the proposed molecular structure. The mass spectra of the Co(II), Ni(II), Rh(III) and Pd(II) chelated forms reveal prominent fragment ion peaks at *m/z* values of 490.93, 526.97, 588.97 and 539.67, respectively. These peaks correspond to the chelated species with the following empirical formulas: CoC_24_H_17_N_2_O_4_Cl, NiC_24_H_21_N_2_O_6_Cl, RhC_24_H_19_N_2_O_5_Cl_2_ and PdC_24_H_17_N_2_O_4_Cl, including molecular weights of 491.79, 527.58, 589.23 and 539.28 g mol^−1^, respectively. The mass spectrum of the Cu(II) chelate (CuC_24_H_17_N_2_O_4_Cl, 496.41 g mol^−1^) did not show an intact molecular ion peak, probably owing to fragmentation during ionization. Instead, a prominent fragment ion ([F]^+^) was observed at *m/z* = 490.38, along with additional fragment ions at *m/z* = 443.28, 372.06, 207.61, 131.84 and 119.87. These fragments are consistent with the proposed fragmentation pathway. Although an intact molecular ion peak was not detected, the fragmentation patterns support the proposed formulation when considered alongside the other spectroscopic and analytical data. The mass fragmentation patterns of the free ligand (H-MND) and all complexes are proposed [48], supporting their proposed structural formulas (supplementary material, scheme S1).

### 3.4. UV–visible spectra and magnetic data

UV–vis electronic spectra of the ligand (H-MND) and its M(II/III) complexes were recorded in DMSO solvent within the range of 200–1100 nm. All results are listed in table S2 in the supplementary material. The electronic spectrum of the unchelated ligand exhibits two distinct transition bands corresponding to $\pi \to \overset{*}{\pi }$ transitions resulting from the conjugated system of *π* electrons and $n\to \overset{*}{\pi }$ transitions, with their absorption peak maxima at 34 482 and 27 322 cm^−1^, respectively [53]. Additionally, a third band is observed at 22 883 cm^−1^, which may be attributed to an intramolecular charge transfer (ICT) [54]. In chelates, the $\pi \to \overset{*}{\pi }$ transition remains unaltered upon complexation [55]. Whereas for $n\to \overset{*}{\pi }$, the peak either shifted or vanished in the spectra of the complexes, suggesting coordination of the ligands to the metal (II/III) ions.

The absorption spectrum of the Co(II) complex revealed two bands at 16 694 and 15 220 cm^−1^, corresponding to d–d transitions and may be associated with the ^4^A_2_ to ^4^T_1_(P) and ^4^A_2_ to ^4^T_1_(F) transitions, respectively. The observed bands, along with the complex's magnetic moment of 4.15 B.M., are consistent with a tetrahedral environment around the Co(II) ion [56,57]. The Ni(II) complex displayed two absorption bands at 11 560 and 19 157 cm^−1^. These bands are assigned to the transitions ^3^A_2_g → ^3^T_1_g(F) (ν_2_) and ^3^A_2_g → ^3^T_1_g(P) (ν_3_). Based on the d^8^ (Tanabe–Sugano) diagram, the resulting ratio of Dq/B' (1.1) versus ν_3_/ν_2_ (1.66) and the calculated ν_1_ transition of 7149 cm^−1^ are in agreement that the Ni(II) ion is in an octahedral environment. A proposed octahedral Ni(II) centre with two unpaired electrons is consistent with the observed transitions and the measured magnetic moment of 3.21 B.M. [58–60]. The electronic spectrum of the Cu(II) chelate showed bands at 10 526 and 19 379 cm^−1^. These bands have been assigned to the ^2^B_2_ → ^2^E and ^2^B_2_ → ^2^A_1_ transitions, respectively. Together with the observed magnetic moment of 1.94 B.M., this suggests a tetragonally distorted tetrahedral geometry [61,62]. Two bands were observed in the spectrum of Rh(III) at 18 115 and 25 000 cm^−1^; these can be assigned to the transitions ^1^A_1_g → ^1^T_1_g and ^1^A_1_g → ^1^T_2_g, respectively. The complex exhibited diamagnetic behaviour, consistent with a proposed strong-field octahedral coordination environment [58,63]. The Pd(II) complex spectrum exhibited an absorption peak at 28 901, which may be attributed to a charge transfer band (C.T). Additionally, peaks at 20 080 and 14 598 cm^−1^ are probably associated with the ^1^A_1_g → ^1^B_1_g and ^1^A_1_g → ^1^A_2_g d-d transitions, respectively. These observations, along with the complex's diamagnetic behaviour, suggest a square-planar geometry, typically associated with dsp^2^ hybridization [64,65].

### 3.5. Thermal (TGA-DSC) analysis

The ligand undergoes four distinct decomposition steps at 109.23, 279.81, 327.78 and 534.99°C, which correspond to the successive loss of C_4_H_5_ + 2CO_2_, C_5_H_9_, C_6_H_2_N and C_5_H_2_N, respectively. The thermograms of the Co(II), Cu(II) and Pd(II) complexes revealed three main stages of thermal decomposition. By contrast, the Ni(II) and Rh(III) complexes decomposed in four steps. A summary of the thermal decomposition steps, including experimental and calculated mass losses and their corresponding assignments, is given in table 2. Complete TGA/DSC results are provided in table S3 in the supplementary material. The first thermal decomposition step of the Ni(II) complex occurs within the range of 75.48–175.39°C. This stage corresponds to the removal of two coordinated water molecules [46], supported by an endothermic DSC peak at *T*_max_ = 76.95°C associated with dehydration. By contrast, the TG curve of the Rh(III) chelate shows initial decomposition between 54.79 and 206.21°C, with a mass loss of 2.980% (calc. 3.054%), which corresponds to the elimination of a coordinated water molecule [66]. The Pd(II) complex exhibits minor thermal events at 31.28 and 63.08°C, which correspond to the *T*_max_ signals observed in the DSC analysis without any significant mass loss in the TG profile. The first actual decomposition step in the TG curve occurs between 163.44 and 239.24°C. This observation indicates that no significant residual solvent or surface moisture is present and that the early DSC peaks are suggested owing to minor phase transitions in the matrix [67]. In all cases, the experimental mass losses and residues closely matched the calculated values, supporting the proposed decomposition pathways [68].

**Table 2.** Summary of TGA data for the free ligand and its synthesized complexes.

compounds (M.Wt) g mol^−1^	TG temp. range (°C)	Wt. loss (%) (found) calc.	lost assignment
H–MND ligand (398.42)	109.23–263.97	(35.330) 35.389	C_4_H_5_ + 2CO_2_
	279.81–325.66	(17.560) 17.318	C_5_H_9_
	327.77–534.95	(21.530) 22.087	C_6_H_2_N
	534.99–597.88	(19.220) 19.075	C_5_H_2_N
[Co(MND)Cl] (491.79)	40.85–119.67	(11.280) 11.386	C_4_H_8_
	161.75–338.64	(25.060) 25.112	C_3_H_6_NO_2_Cl
	340.97–546.88	(39.820) 40.871	C_14_H_3_NO
[Ni(MND)(H_2_O)_2_Cl] (527.58)	75.48–175.39	(16.444) 16.490	2H_2_O + C_4_H_3_
	176.97–268.87	(12.263) 12.036	CO + Cl
	296.4–405.29	(32.578) 32.412	C_10_H_7_N_2_O
	446.97–579.58	(18.654) 18.385	C_6_H_9_O
[Cu(MND)Cl] (496.41)	53.68–119.78	(16.010) 16.014	C_3_H_8_ + Cl
	127.95–331.89	(37.240) 37.267	C_10_H_5_N_2_O_2_
	336.79–572.89	(34.10) 33.842	C_11_H_4_O_2_
[Rh(MND)(H_2_O)Cl_2_] (589.23)	54.79–206.21	(2.980) 3.054	H_2_O
	210.08–327.45	(17.479) 17.310	2Cl + OCH_3_
	329.14–352.56	(36.350) 36.997	C_15_H_8_NO
	356.91–557.12	(23.489) 23.080	C_7_H_6_NO_2_
[Pd(MND)Cl] (539.28)	163.44–239.24	(43.454) 43.298	C_11_H_6_N_2_O_2_Cl
	242.21–382.71	(13.079) 12.609	C_5_H_8_
	382.71–575.59	(19.318) 19.099	C_7_H_3_O

## 4. *In vitro* biological evaluation

### 4.1 Antioxidant activity

The antioxidant activity of the newly synthesized ligand and its metal chelates was evaluated using the DPPH radical-scavenging assay (supplementary material, table S4). All compounds exhibited concentration-dependent radical-scavenging activity, and their effectiveness was compared with that of the standard antioxidants, gallic acid and Trolox, under identical experimental conditions. Among the tested compounds, the Ni(II) complex exhibited strong antioxidant efficacy with an IC_50_ of 22.89 ± 0.28 µg ml^−1^, lower than that of Trolox (figure 1). The IC_50_ value for Ni(II) complex was significantly different from that of gallic acid (IC_50_ = 21.58 ± 0.54 µg ml^−1^). This difference was confirmed by an independent samples *t*-test (*t* = 4.31, *p* ˂ 0.01). Furthermore, the Ni(II) complex demonstrated improved radical-scavenging activity compared with its free ligand and other metal complexes. While the specific mechanism was not examined in this study, the improved activity may be attributed to metal coordination affecting the electronic properties of the ligand framework. This influence could facilitate hydrogen atom donation and/or electron-transfer processes crucial to DPPH scavenging. Similar patterns have been reported in Ni(II)-based complexes, in which metal coordination enhances antioxidant activity by modulating the ligand's electronic environment and stabilizing radical intermediates [69,70]. These observations suggest a possible structure-activity relationship for the Ni(II) complex; however, further mechanistic studies are needed to confirm this interpretation.

**Figure 1.**
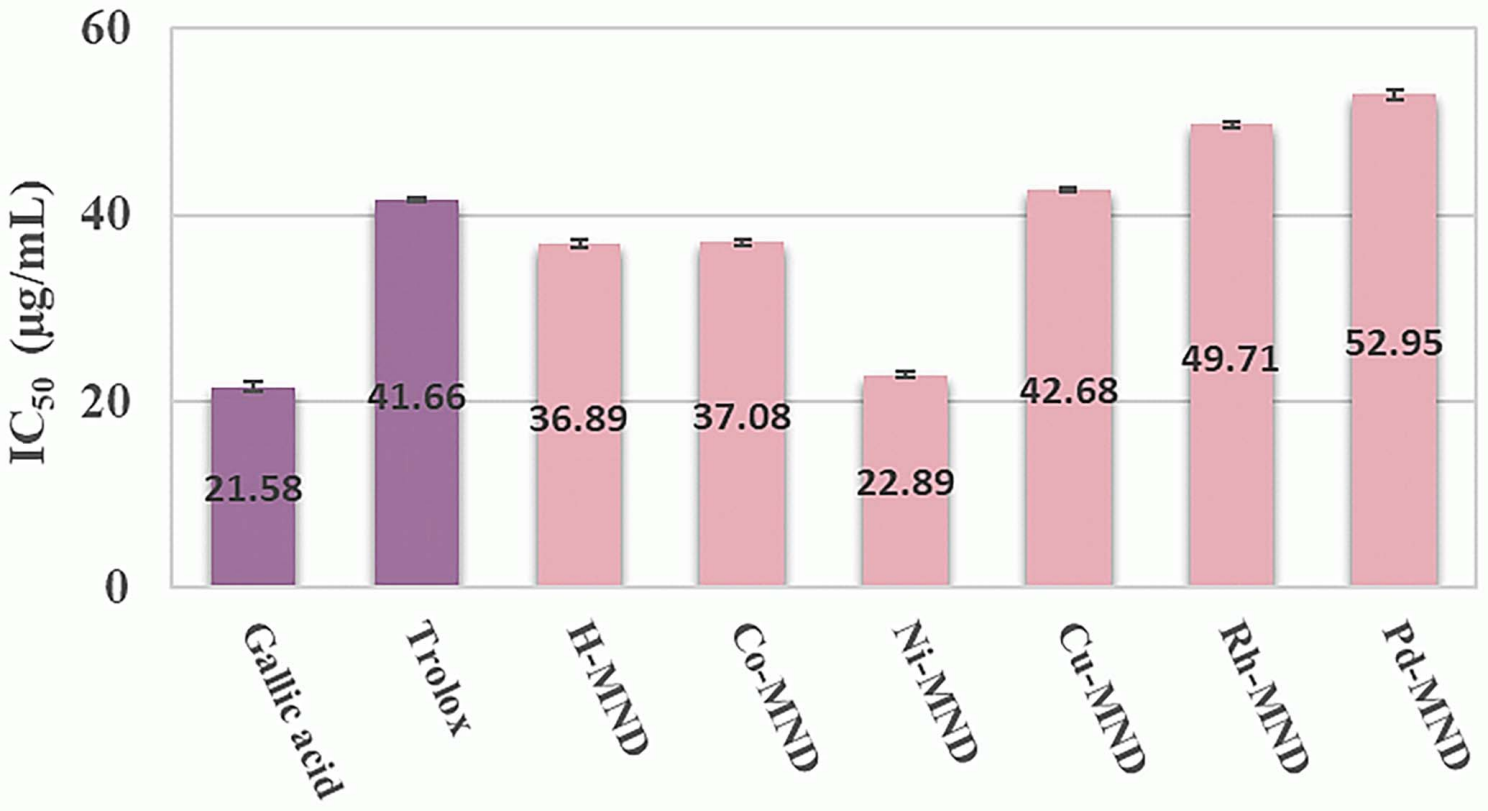
The *in vitro* DPPH scavenging potency (IC_50_, µg ml^−1^) of the ligand and its complexes; mean ± SD, *n* = 4.

### 4.2. Plasmid DNA cleavage studies

The DNA-cleavage activity of the free ligand and its metal complexes was assessed using the pTXB1 plasmid DNA as the target substrate. As illustrated in figure S8 in the supplementary material, the untreated plasmid DNA (lane P) displayed a distinct band corresponding to intact plasmid pTXB1 DNA. The free ligand, along with most of the examined Co(II), Ni(II) and Rh(III) metal complexes, resulted in electrophoretic patterns similar to those of the untreated control, with no observable changes in plasmid DNA mobility or band distribution under the experimental conditions employed. By contrast, treatment with the Pd(II) complex resulted in the appearance of an additional DNA band, suggesting partial conversion of the native plasmid into an alternative topological form, possibly reflecting DNA cleavage activity. Notably, the reaction occurred in the absence of externally added oxidizing or reducing agents.

The exclusive DNA-modifying activity observed in the Pd(II) complex may be associated with its stronger DNA interactions compared with the other complexes. Differences in DNA cleavage efficiency among structurally similar metal complexes are often attributed to variations in their DNA-binding affinity and modes of DNA interaction [71,72]. Although the marked alteration in plasmid DNA topology suggests partial DNA cleavage, the precise nature of this process cannot be established from the present data.

### 4.3. Phenoxazinone synthase (mimicking activity)

The biomimetic catalytic behaviour of the copper and cobalt complexes was investigated by monitoring the aerobic oxidation of OAP to phenoxazinone (APX) in DMSO. The reaction progress was validated by a time-dependent increase in absorbance at 436 nm following the addition of the substrate (figure 2).

**Figure 2.**
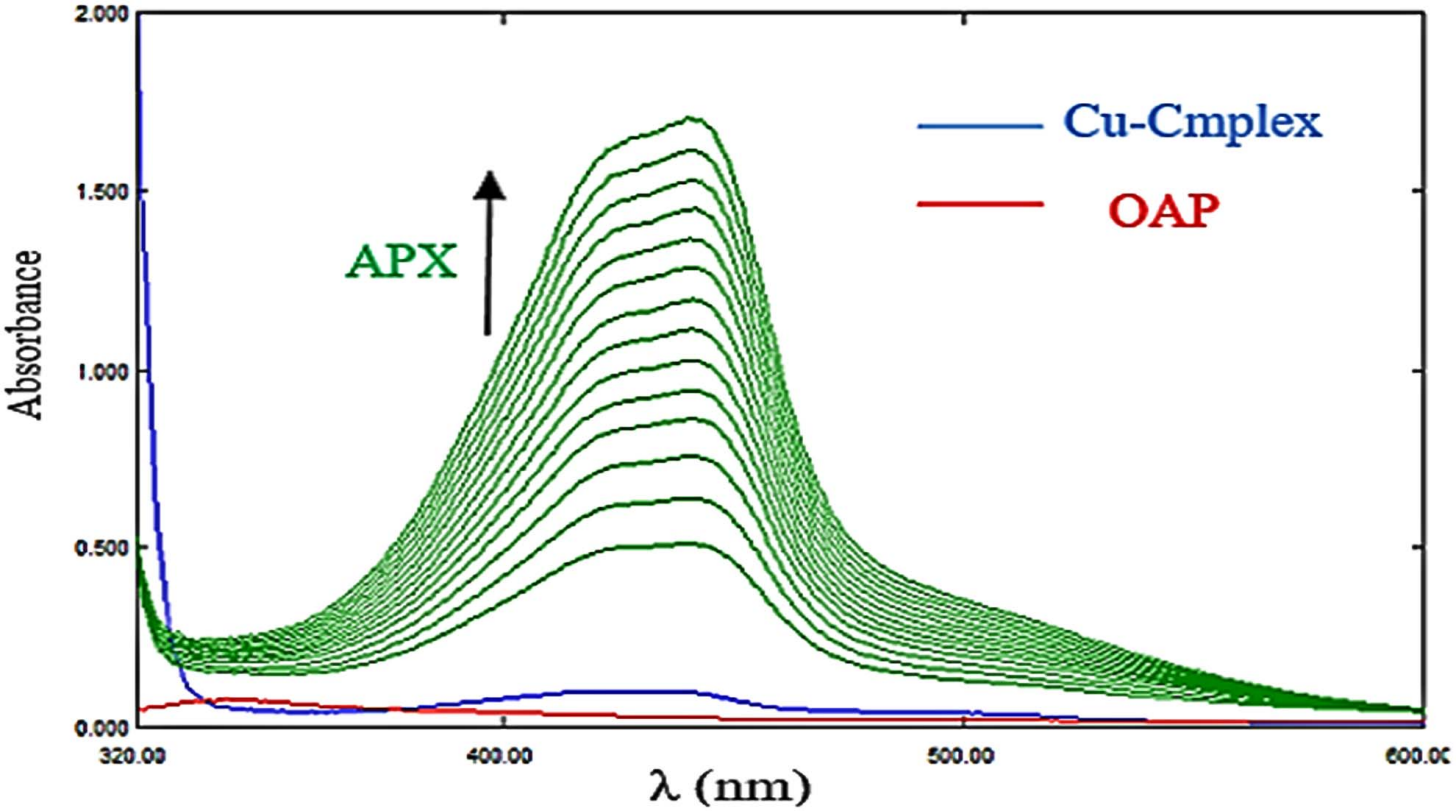
The time-dependent spectral development of 2-aminophenoxazine-3-one (APX) upon addition of 10^−2^m substrate (OAP) to 3 × 10^−5^m of Cu(II) complex 5 min after each reading cycle.

The catalytic activity was evaluated by spectrophotometry, and kinetic studies were performed to characterize the catalytic behaviour of the complexes. Solutions of Cu(II) and Co(II) complexes were prepared at initial concentrations of 3 × 10^−5^ and 6 × 10^−5^m, respectively, and reacted with varying concentrations of OAP for this purpose. Initial rate versus substrate concentration plots (figure 3) showed saturation kinetics for both complexes, consistent with Michaelis–Menten kinetics and the formation of a catalyst-substrate complex prior to oxidation. Michaelis–Menten plots, along with their corresponding Lineweaver–Burk representations (inset), are illustrated in figure 3. Table 3 summarizes the apparent kinetic parameters obtained for the phenoxazinone synthase-like activity of the investigated complexes. The Cu(II) and Co(II) systems produced *k*_cat_ values of 82.30 ± 2.89 and 0.286 ± 0.011 min^−1^, respectively, under the experimental conditions employed. These parameters represent the catalytic behaviour of each complex under its respective assay conditions and catalyst concentration.

**Figure 3.**
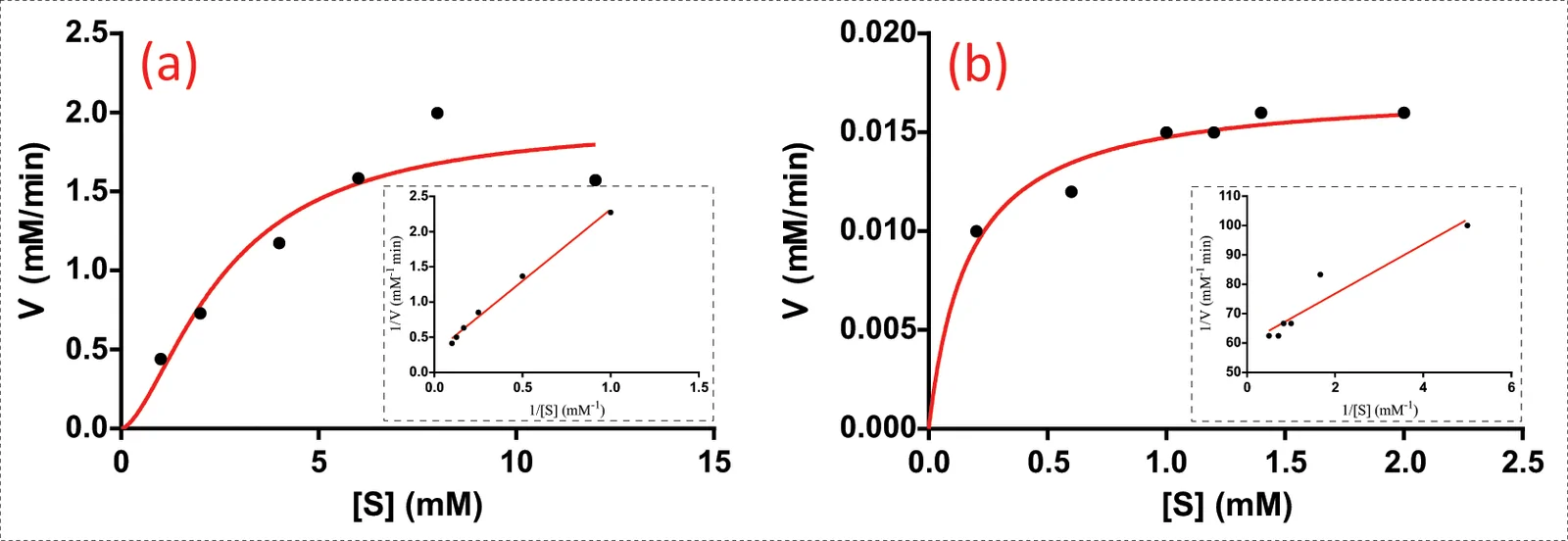
Plot of the rate for the aerial oxidation catalyzed by (a) Cu(II) / (b) Co(II) complexes versus (OAP) as [substrate]; [insert: Lineweaver–Burk graphics].

**Table 3.** Apparent kinetic parameters for the phenoxazinone synthase-like (PXS-like) activity of the investigated complexes under their respective experimental conditions.

mean ± s.d. (*n* = 4)					
compounds	*V*_0_ ( m min^−^^1^)	*V*_max_ ( m min^−^^1^)	*K*_M_ ( m)	*k*_cat_ (min^−^^1^)	*k*_cat_/K_M_ ( m^−^^1^ min^−^^1^)
[Co(MND)Cl]	(1.60 ± 0.05) × 10^−5^	(1.72 ± 0.06) × 10^−5^	(1.68 ± 0.08) × 10^−4^	0.286 ± 0.011	(0.17 ± 0.01) × 10^4^
[Cu(MND)Cl]	(1.57 ± 0.06) × 10^−3^	(2.469 ± 0.09) × 10^−3^	(3.97 ± 0.16) × 10^−3^	82.30 ± 2.89	(2.07 ± 0.11) × 10^4^

The observed catalytic behaviour, depending on the specific system, may be influenced by differences in the electronic structures and coordination environments of the metal centres involved. Variations in structural flexibility and electronic properties across coordination environments have been extensively documented in the literature [73], and these differences may impact catalytic performance in related systems. Similar effects have been noted in related bio-inspired systems, such as copper and cobalt scorpionate frameworks [74]. The generation of aminophenoxazinone (APX) from OAP, with the catalytic action of copper and cobalt complexes, was observed only in the presence of molecular oxygen. In the absence of O_2_, the APX chromophore is not detected, a finding consistent with previous research [36]. According to earlier studies, the aerobic oxidation of o-aminophenol proceeds via an oxidative pathway involving molecular dioxygen, consistent with established reports of similar catalytic oxidation processes [75,76]. A plausible reaction pathway for the oxidation of o-aminophenol systems is proposed in scheme 2, which summarizes previous studies [24,76].

**Scheme 2.**
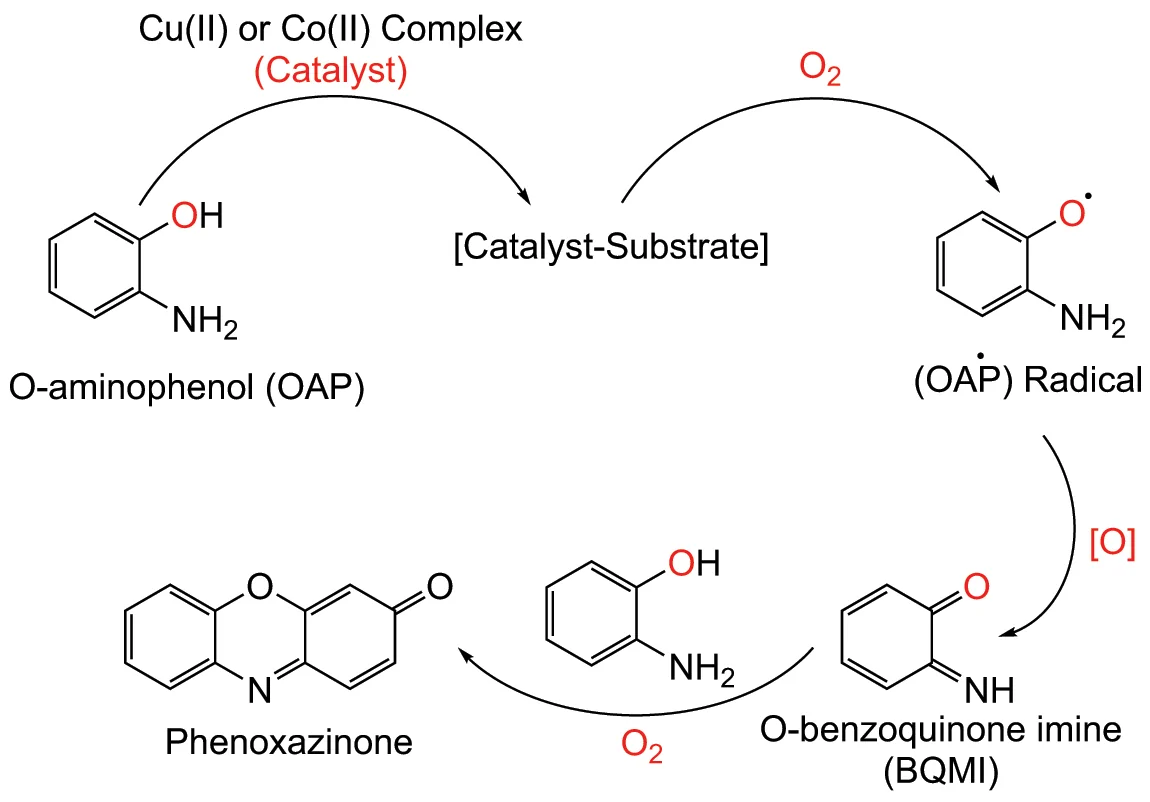
Proposed mechanism of o-aminophenol via the aerial oxidation route.

## 5. Conclusion

A new anthraquinone-eugenol-based azo ligand and its chelates with Co(II), Ni(II), Cu(II), Rh(III) and Pd(II) were successfully synthesized. The proposed ONO tridentate coordination mode of the ligand is supported by the collective spectroscopic and analytical evidence, with the ^1^H- and ^13^C-NMR data for the Pd(II) complex providing further insights into the ligand's coordination behaviour. However, the absence of single-crystal X-ray diffraction data excludes conclusive verification of the structures. *In vitro* biological studies demonstrated multi-functional properties of these complexes. Notably, the Ni(II) complex demonstrated the highest antioxidant activity among the compounds studied, exhibiting strong radical scavenging efficiency relative to the reference antioxidants gallic acid and Trolox. Also, the Pd(II) complex was the only compound in the series to exhibit plasmid DNA-modifying activity consistent with partial cleavage without requiring external redox agents, highlighting the critical influence of the metal centre on this biological activity. The Cu(II) complex showed a *k*_cat_ value of 82.30 ± 2.89 min^−1^ in the PHS-like system under the given experimental conditions. These findings underscore the importance of ligand design and metal coordination in modulating bioactivity, suggesting that anthraquinone-eugenol-based azo-metal complexes warrant further investigation. Additional research is needed to evaluate their biological properties and explore their potential applications.

## Supplementary Material



## Data Availability

The data that support the findings of this study are available in the supplementary material of this article [77].
