# Supplementary material for: Synthesis, spectroscopic study, antioxidant, DNA-cleavage and enzyme-mimicking activities for metal(II/III) complexes with azo ligand–eugenol scaffold
Source: R Soc Open Sci. 2026 Oct 21;13(10):252298. doi: 10.1098/rsos.252298 (PMC13701512; doi:10.1098/rsos.252298)
Supplement: Supplementary file 1 [file rsos.252298.zip › rsos.252298/rsos252298_si_001.pdf]

Supporting Information

Synthesis, Spectroscopic Study, Antioxidant, DNA-Cleavage and Enzyme-Mimicking Activities for Metal$^{(II/III)}$ Complexes with Azo Ligand-Eugenol Scaffold

Huda Muayad Nafea*[1,2]. Abbas Ali Salih Al-Hamdani[2]

[1] Department of Chemistry, College of Science, University of Baghdad, Baghdad, Iraq
[2] Department of Chemistry, College of Science for Women, University of Baghdad, Baghdad, Iraq

Experimental Section

Synthesis of 1-(5-allyl-2-hydroxy-3-methoxyphenyl)diazenyl)anthracene-9,10-dione ligand (H-MND)

An ice bath was used to cool a well-agitated solution of 1-aminoanthraquinone (0.669 g, 3.00 mmol) in 5 mL of sulfuric acid. In a separate round-bottom flask, 0.207 g (3.00 mmol) of $NaNO_2$ was dissolved in 2 mL of concentrated sulfuric acid. The cooled amine mixture was then added to the flask containing the nitrosonium composition. After stirring for 4 hours at a temperature below 5 °C, the reaction mixture was added to 50 mL of cold water and stirred for an additional 30 minutes. The diazonium salt was separated from the reaction mixture by filtration. Next, a cold solution of eugenol (0.50 g, 3.00 mmol) in an aqueous NaOH solution was mixed dropwise with the resultant diazonium salt solution, continuously stirred for another hour at a temperature between (0-5) °C. Finally, the mixture was filtered and washed with distilled water and diethyl ether. The product was dried in a desiccator using anhydrous $CaCl_2$.

Synthesis of metal complexes

The metal chelates were synthesized by mixing 1.00 mmol of metal salts ($CoCl_2{\cdot}6H_2O$, $NiCl_2{\cdot}6H_2O$, $CuCl_2{\cdot}2H_2O$, $RhCl_3{\cdot}3H_2O$, and $PdCl_2$) with 1.00 mmol of ligand in hot absolute ethanol. The mixture was then refluxed for different durations: 5 hours for nickel, rhodium, and

palladium complexes, and 3 hours and 7 hours for cobalt and copper complexes, respectively. After refluxing, the mixture was cooled. The progress of the reaction was monitored using thin-layer chromatography (TLC). The precipitate was then filtered, washed with hot ethanol and diethyl ether, and dried with anhydrous $CaCl_2$ in a desiccator.

Biological methods (*In Vitro*)

Antioxidant method

Solutions of the compounds and standards (Gallic acid and Trolox) were prepared at concentrations ranging from 12.5 to 200 µg/mL, using a stock solution starting from a 1.00 mg/mL solution in dimethyl sulfoxide. Aliquots of 0.1 mL were mixed with the DPPH solution (3.9 mL), incubated at 37 °C for 30 minutes, and then cooled. The absorbance was recorded at 517 nm. GraphPad Prism software was used to develop the graphs and calculate the IC_{50} values (the concentration of the sample required to achieve 50% inhibition) from the DPPH data.

Plasmid DNA-Cleavage Experiment

The analysis was evaluated against pTXB1 plasmid DNA via agarose gel electrophoresis. Agarose (250) mg was dissolved in 25 mL of Tris-Borate-EDTA buffer (TBE) by heating, cooled to ~55 °C, and cast into a gel cassette. After solidification, the gel was placed in an electrophoresis chamber containing TBE buffer, and test compounds were dissolved in freshly distilled DMSO (1.0 mg mL^{-1}). In the DNA cleavage assay, each compound was incubated separately with pTXB1 plasmid DNA for 2 hours at 37 °C. All reaction mixtures were analyzed on the same agarose gel alongside an untreated plasmid control and a 1 kb DNA marker. After incubation, 20 µL of each sample was mixed with bromophenol blue loading dye in a 1:1 (v/v) ratio and subjected to electrophoresis at 50 V for approximately 45 minutes. The gel was then stained with ethidium bromide (10 µg/mL) for 10-15 min, visualized under a UV transilluminator and photographed. DNA cleavage activity was evaluated by comparing the results with the untreated control.

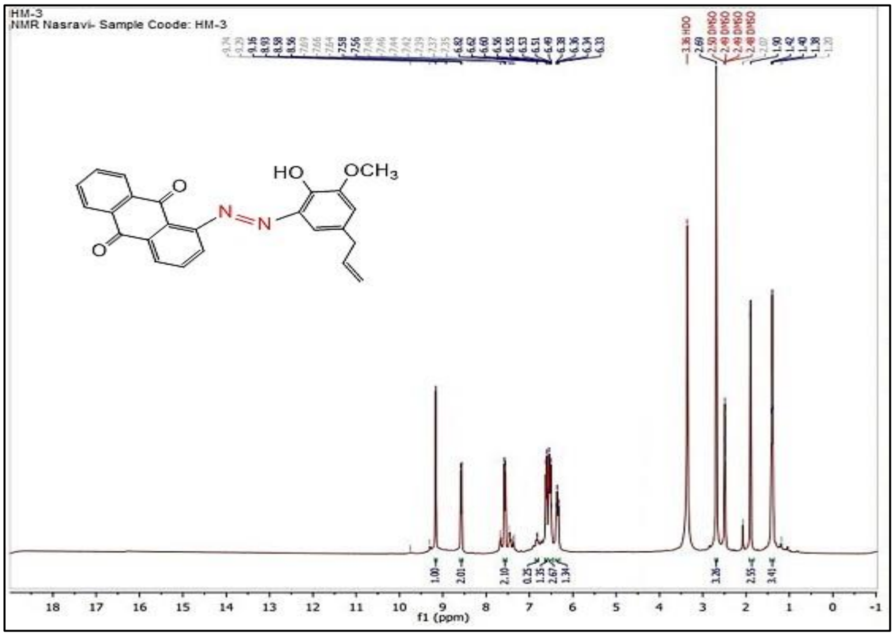

Figure S1. ^{1}H-NMR spectrum of free ligand in DMSO-d_6

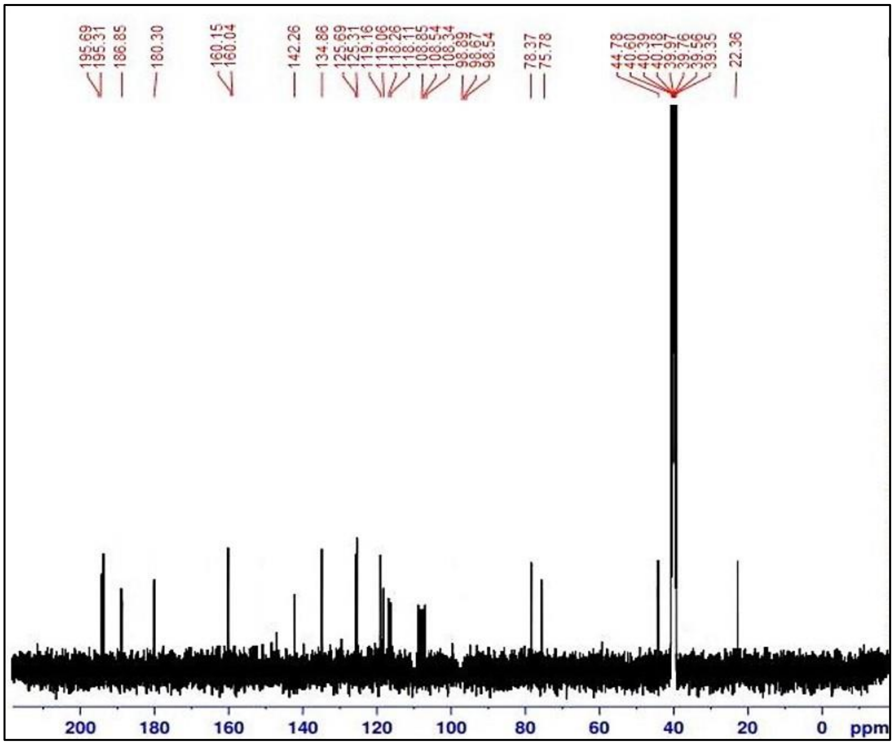

Figure S2. ^{13}C-NMR spectrum of free ligand in DMSO-d_6

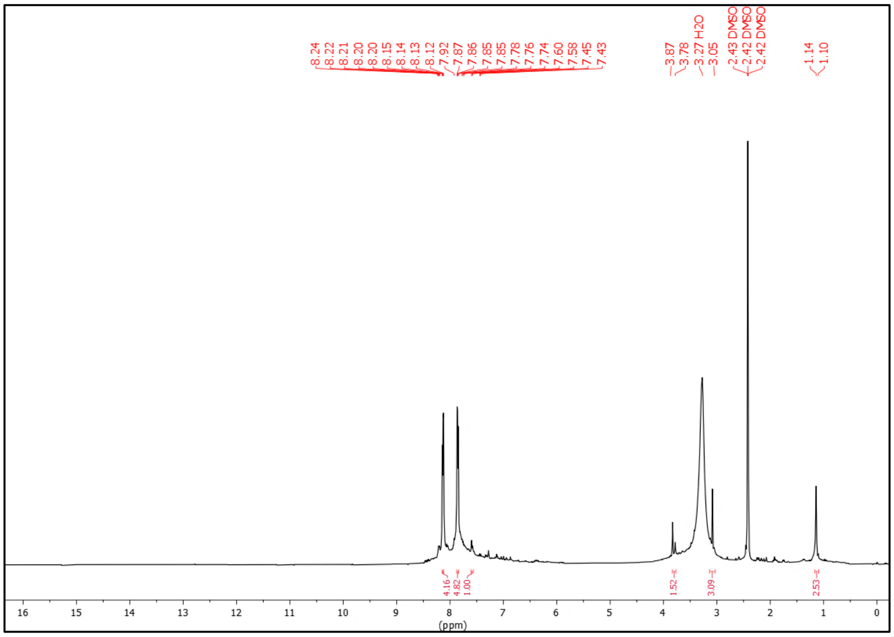

Figure S3. ^{1}H-NMR spectrum of Pd(II) complex in DMSO-d_6

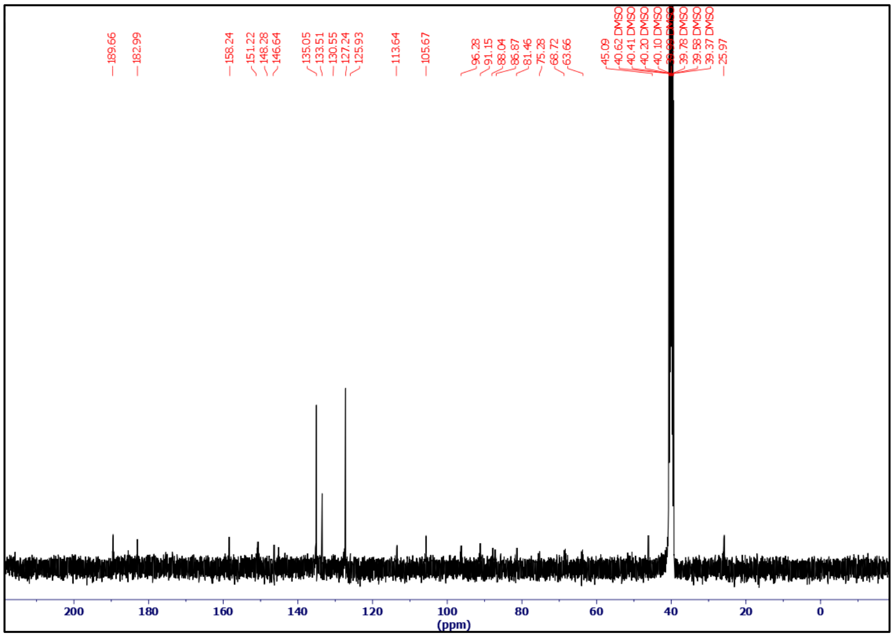

Figure S4. ^{13}C-NMR spectrum of Pd(II) complex in DMSO-d_6

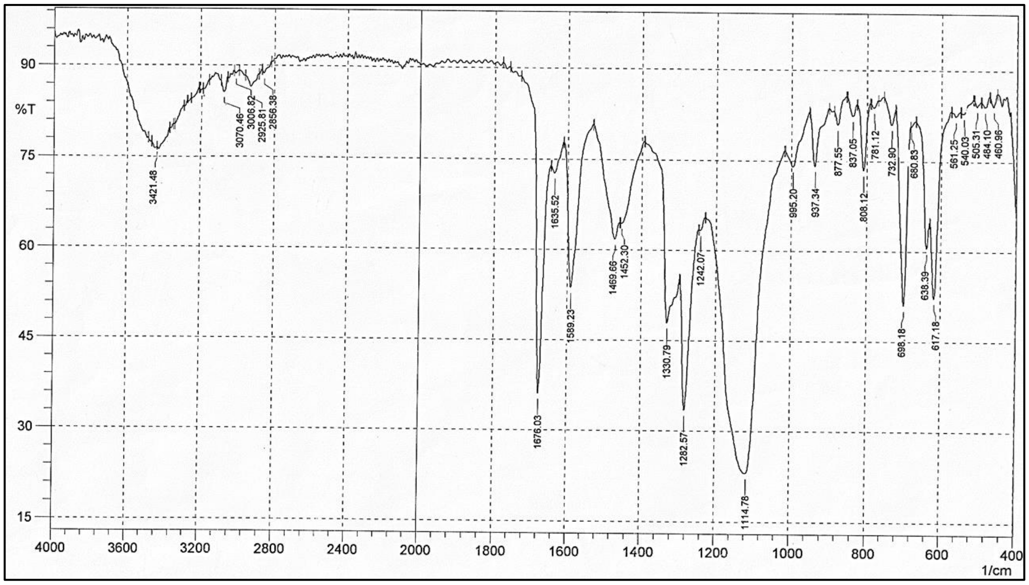

Figure S5. FT-IR spectrum of free ligand.

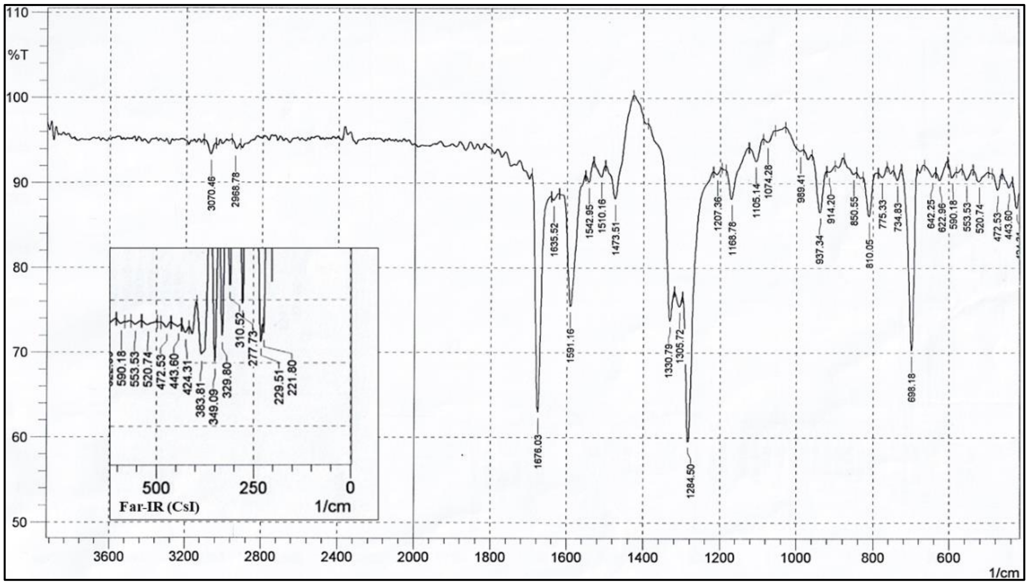

Figure S6. FT-IR spectrum of Co(II) complex (KBr); Inset: Far-IR region (CsI).

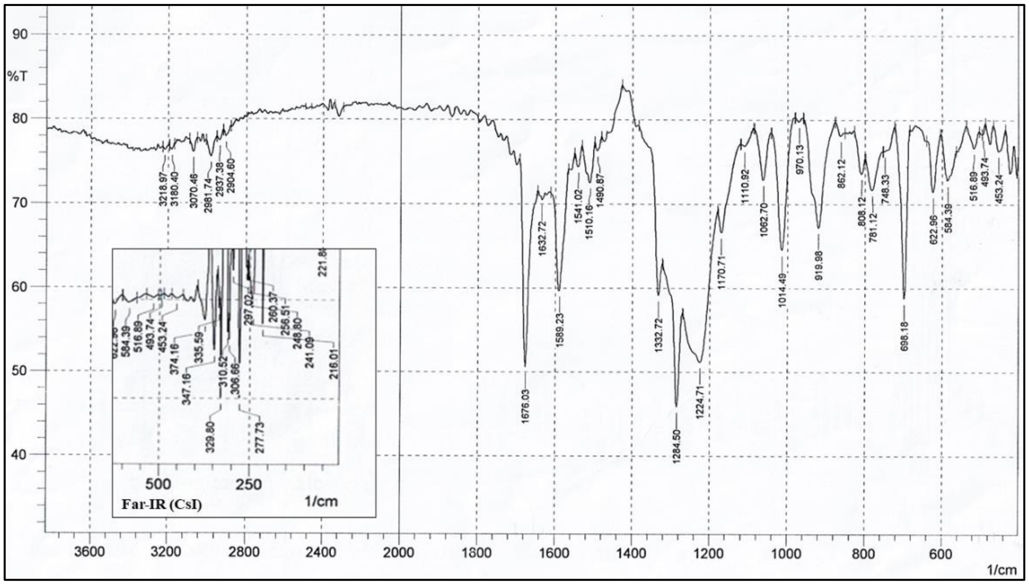

Figure S7. FT-IR spectrum of Pd(II) complex (KBr); Inset: Far-IR region (CsI).

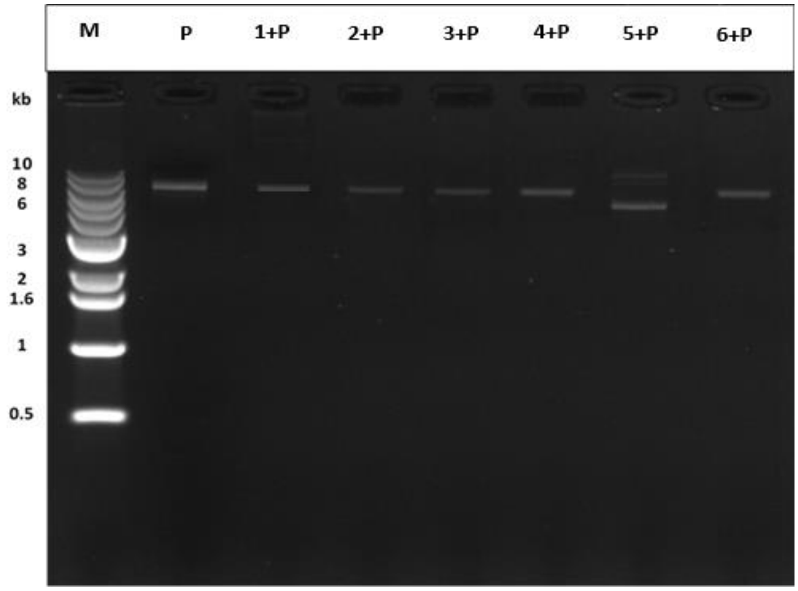

Figure S8. DNA-cleavage (gel electrophoresis pattern) of pTXB1 plasmid DNA incubated with the synthesized compounds. Lane M: DNA ladder; Lane P: untreated pTXB1 plasmid DNA control; Lane 1+P: plasmid + free ligand; Lane 2+P: plasmid + Co(II) complex; Lane 3+P: plasmid + Ni(II) complex; Lane 4+P: plasmid + Cu(II) complex; Lane 5+P: plasmid + Pd(II) complex; Lane 6+P: plasmid + Rh(III) complex;

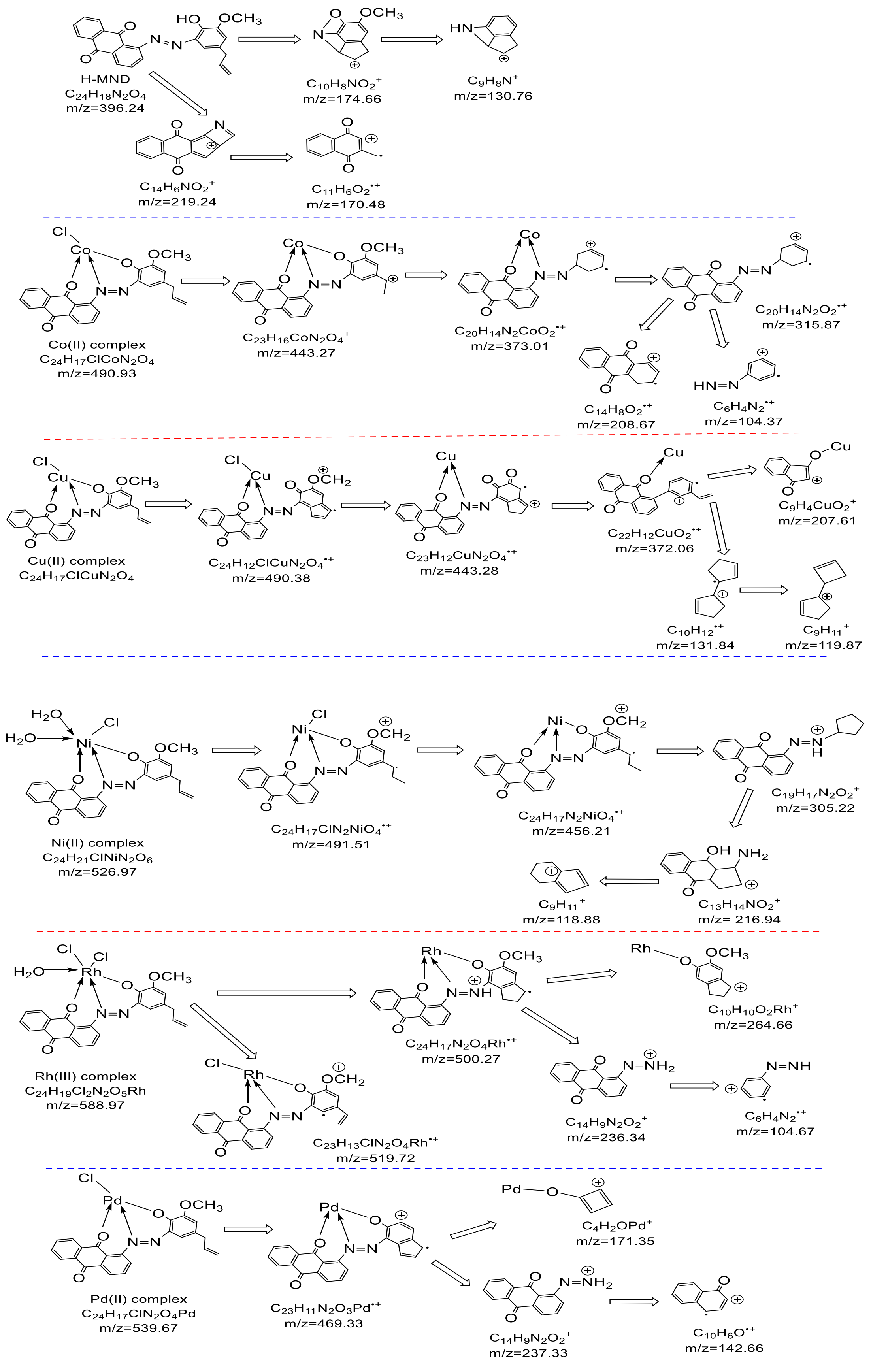

Scheme S1. The proposed mass fragmentation pattern of the ligand (H-MND) and its chelates.

Table S1. The main indication of the infrared bands in the ligand (H-MND) and its complexes (cm^{-1}).

Compounds	ν(OH)phenolic<br>ν(C-O) phenolic	ν(H_2O) aqua<br>ρr<br>ρw	ν(N=N)	ν(C=O)	ν(allyl C=C/C=O overlap)	New band (chelate framework)	ν(M-O)	ν(M-N)	ν(M-Cl)
H-MND	3421<br>1114	–	1452<br>1469	1676	1635	–	–	–	–
[Co(MND)Cl]	–<br>1168	–	1510<br>1473	1676	1635	1542	590	472	329
[Ni(MND)(H_2O)$_2$Cl]	–<br>1168	3396<br>889<br>624	1510<br>1475	1676	1625	1544	580	449	270
[Cu(MND)Cl]	–<br>1168	–	1519<br>1471	1676	1641	1542	590	522	306
[Rh(MND)(H_2O)Cl_2]	–<br>1170	3373<br>860<br>655	1508<br>1483	1676	1622	1544	594	437	325
[Pd(MND)Cl]	–<br>1170	–	1510<br>1490	1676	1632	1541	584	453	335

Table S2. The electronic spectral and magnetic data of the ligand (H-MND) and chelated compounds.

Compounds	λ (nm)	Absorption bands (cm^{-1})	Transition Character	μ_{eff} (B.M.)	Proposed Geometry
H-MND	290	34482	π→π*	–	–
	366	27322	n→π*		
	437	22883	ICT		
[Co(MND)Cl]	284	35211	π→π*	4.15	Tetrahedral
	325	30769	n→π*		
	599	16694	$^4A_2 \rightarrow ^4T_{1\,(P)}$		
	657	15220	$^4A_2 \rightarrow ^4T_{1\,(F)}$		
[Ni(MND)(H_2O)$_2$Cl]	286	34965	π→π*	3.21	Octahedral
	318	31446	n→π*		
	522	19157 ν_3	$^3A_2g \rightarrow ^3T_1g\,_{(P)}$		
	865	11560 ν_2	$^3A_2g \rightarrow ^3T_1g\,_{(F)}$		
	1398	7149 (calc.) ν_1	$^3A_2g \rightarrow ^3T_2g\,_{(F)}$		
[Cu(MND)Cl]	288	34722	π→π*	1.94	Distorted Tetrahedral
	297	33670	n→π*		
	516	19379	$^2B_2 \rightarrow ^2A_1$		
	950	10526	$^2B_2 \rightarrow ^2E$		
[Rh(MND)(H_2O)Cl_2]	295	33898	π→π*	Diam	Octahedral
	326	30674	n→π*		
	400	25000	$^1A_{1g} \rightarrow ^1T_{2g}$		
	552	18115	$^1A_{1g} \rightarrow ^1T_{1g}$		
[Pd(MND)Cl]	288	34722	π→π*	Diam	Square planar
	309	32362	n→π*		
	346	28901	C.T		
	498	20080	$^1A_{1g} \rightarrow ^1B_{1g}$		
	685	14598	$^1A_{1g} \rightarrow ^1A_{2g}$		

Table S3. The results analysis of TGA-DSC for the ligand (H-MND) and metal complexes.

TGA Data					DSC Data		
Compounds Molecular formula (M.Wt) g./mol	TG Temp. range (°C)	Wt. loss (%) (found) Calc.	Lost assignment	Residuals (%) (found) Calc.	T_i - T_f (°C)	T_{max} (°C)	ΔH (J/g)
H-MND ligand $C_{24}H_{18}N_2O_4$ (398.42)	109.23-263.97	(35.330) 35.389	$C_4H_5 + 2CO_2$	2C (6.360) 6.028	155.36-159.31	157.53	− 0.39
	279.81-325.66	(17.560) 17.318	C_5H_9		313.14-319.44	315.37	−3.29
	327.77-534.95	(21.530) 22.087	C_6H_2N				
	534.99-597.88	(19.220) 19.075	C_5H_2N				
[Co(MND)Cl] $C_{24}H_{17}ClCoN_2O_4$ (491.79)	40.85-119.67	(11.280) 11.386	C_4H_8	CoO +3C (23.840) 22.562	66.28-125.75	99.29	−112.68
	161.75-338.64	(25.060) 25.112	$C_3H_6NO_2Cl$				
	340.97-546.88	(39.820) 40.871	$C_{14}H_3NO$				
[Ni(MND)(H_2O)$_2$Cl] $C_{24}H_{21}ClNiN_2O_6$ (527.58)	75.48-175.39	(16.444) 16.490	$2H_2O + C_4H_3$	NiO + 3C (20.061) 20.986	71.38-79.69	76.95	−1.69
	176.97-268.87	(12.263) 12.036	CO + Cl		120.57-128.39	124.85	− 0.64
	296.4-405.29	(32.578) 32.412	$C_{10}H_7N_2O$		291.05-299.68	296.67	− 3.12
	446.97-579.58	(18.654) 18.385	C_6H_9O				
[Cu(MND)Cl] $C_{24}H_{17}ClCuN_2O_4$ (496.41)	53.68-119.78	(16.010) 16.014	C_3H_8 + Cl	Cu (12.650) 12.801	79.29-85.21	81.12	−1.94
	127.95-331.89	(37.240) 37.267	$C_{10}H_5N_2O_2$		133.12-139.91	135.43	−1.94
	336.79-572.89	(34.10) 33.842	$C_{11}H_4O_2$		291.03-298.43	295.37	−3.38
[Rh(MND)(H_2O)Cl_2] $C_{24}H_{19}Cl_2N_2O_5Rh$ (589.23)	54.79-206.21	(2.980) 3.054	H_2O	Rh + C (19.702) 19.500	41.08-106.52	66.81	−38.85
	210.08-327.45	(17.479) 17.310	$2Cl + OCH_3$		127.85-172.88	150.79	−21.38
	329.14-352.56	(36.350) 36.997	$C_{15}H_8NO$		182.36-196.21	189.75	−1.19
	356.91-557.12	(23.489) 23.080	$C_7H_6NO_2$		218.64-305.41	254.77	241.78
[Pd(MND)Cl] $C_{24}H_{17}ClN_2O_4Pd$ (539.28)	163.44-239.24	(43.454) 43.298	$C_{11}H_6N_2O_2Cl$	PdO + C (24.149) 24.923	14.95-43.28	31.28	−30.84
	242.21-382.71	(13.079) 12.609	C_5H_8		43.34-84.63	63.08	−3.00
	382.71-575.59	(19.318) 19.099	C_7H_3O		164.52-247.82	217.32	−128.07

Table S4. Antioxidant activity data using the DPPH method for the compounds.

Compound	Concentration (μg/mL)					IC_{50} (μg/mL)
	200	100	50	25	12.5	
	Radical Scavenging Activity % (mean ± SD), n = 4					
Gallic acid	91.31±0.44	84.12±0.23	79.65±0.52	63.47±0.91	49.29±0.95	21.58±0.54
Trolox	82.54±0.54	72.48±0.44	66.74±0.62	57.11±0.49	41.65±0.17	41.66±0.19
H-MND	88.65±0.19	80.72±0.65	75.64±0.54	59.74±0.41	46.20±0.42	36.89±0.41
Co-MND	87.64±0.11	81.61±0.49	73.62±0.32	57.98±0.47	44.42±0.19	37.08±0.32
Ni-MND	90.12±0.24	81.61±0.54	74.46±0.47	59.88±0.74	47.94±0.37	22.89±0.28
Cu-MND	79.65±0.22	69.54±0.44	55.64±0.24	49.64±0.36	38.64±0.15	42.68±0.21
Rh-MND	75.11±0.41	67.41±0.12	52.61±0.24	45.45±0.17	39.64±0.55	49.71±0.23
Pd-MND	71.12±0.32	61.51±0.51	51.64±0.44	42.64±0.47	37.61±0.41	52.95±0.48
